# Antibacterial and Antioxidant Capacities and Attenuation of Lipid Accumulation in 3T3-L1 Adipocytes by Low-Molecular-Weight Fucoidans Prepared from Compressional-Puffing-Pretreated *Sargassum Crassifolium*

**DOI:** 10.3390/md16010024

**Published:** 2018-01-11

**Authors:** Chun-Yung Huang, Chia-Hung Kuo, Chia-Hsin Lee

**Affiliations:** Department of Seafood Science, National Kaohsiung Marine University, No. 142, Haijhuan Rd., Nanzih District, Kaohsiung 81157, Taiwan; chkuo@webmail.nkmu.edu.tw (C.-H.K.); j58675@yahoo.com.tw (C.-H.L.)

**Keywords:** 3T3-L1 cells, antibacterial, antioxidant, lipid accumulation, low-molecular-weight fucoidan, *Sargassum crassifolium*

## Abstract

In this study, we extracted fucoidan from compressional-puffing-pretreated *Sargassum crassifolium* by hot water. The crude extract of fucoidan (SC) was degraded by various degradation reagents and four low-molecular-weight (LMW) fucoidans, namely SCO (degradation by hydrogen peroxide), SCA (degradation by ascorbic acid), SCOA (degradation by hydrogen peroxide + ascorbic acid), and SCH (degradation by hydrogen chloride) were obtained. The degradation reagents studied could effectively degrade fucoidan into LMW fucoidans, as revealed by intrinsic viscosity, agarose gel electrophoresis, and molecular weight analyses. These LMW fucoidans had higher uronic acid content and sulfate content than those of SC. It was found that SCOA exhibited antibacterial activity. All LMW fucoidans showed antioxidant activities as revealed by DPPH (2,2-diphenyl-1-picrylhydrazyl), ABTS (2,2′-azino-bis(3-ethylbenzothiazoline-6-sulfonic acid) diammonium salt), and FRAP (ferric reducing antioxidant power) methods. Biological experiments showed that SC and SCOA had relatively high activity for the reversal of H_2_O_2_-induced cell death in 3T3-L1 adipocytes, and SCOA showed the highest effect on attenuation of lipid accumulation in 3T3-L1 adipocytes. Therefore, for the LMW fucoidans tested, SCOA showed antibacterial activity and had a high fucose content, high sulfate content, high activity for the reversal of H_2_O_2_-induced cell death, and a marked effect on attenuation of lipid accumulation. It can thus be recommended as a natural and safe antibacterial and anti-adipogenic agent for food, cosmetic, and nutraceutical applications.

## 1. Introduction

Antibacterial agents are synthetic or natural compounds that interfere with the growth and division of bacteria. A variety of studies have shown that pathogenic microorganisms in humans and various animal species have developed resistance to drugs. This drug resistance is due to the random or otherwise inappropriate usage of commercial antimicrobial agents. As such, there is an urgent need for new antibacterial agents. In addition, synthetic antibiotics have been known to induce side effects such as the appearance of resistant bacteria, skin irritation, organ damage, and immunohypersensitivity [1]. Accordingly, there is growing interest in the development of new agents, especially compounds derived from natural sources, with higher antibacterial activity but with lower or possibly even no side effects [2].

Oxidative stress is defined as the result of the net amount of reactive oxygen species (ROS) exceeds the antioxidant capacity, which can take place as a consequence of a general increase in ROS generation, a depression of the antioxidant systems, or both [3]. ROS in the forms of superoxide anion radical (O_2_^−•^), hydroxyl radical (^•^OH), hydrogen peroxide (H_2_O_2_), singlet oxygen (^1^O_2_), and nitric oxide (NO^•^) are metabolic products and which may also be present in the environment [4]. The excessive production of ROS may damage cellular DNA, proteins, and lipids, leading to either alteration of biochemical compounds, or corrosion of cell membranes [5]. Antioxidants are substances that delay or prevent the oxidation of cellular oxidizable substrates. They exert their effect by scavenging ROS or preventing the generation of ROS [6]. Synthetic antioxidant compounds such as butylated hydroxytoluene (BHT) and butylated hydroxyanisole (BHA) have potent antioxidant activity and are commonly used in processed foods. However, they have been restricted because of their carcinogenicity and other toxic properties [7,8]. Therefore, recent research efforts have been attempted to develop naturally derived antioxidants, particularly those which possess potential nutritional and therapeutic value. Lipid accumulation in adipocytes due to impaired lipid metabolism causes adipocyte hypertrophy and accumulation of adipose tissue, which lead to obesity and metabolic syndrome [9,10]. In vitro and in vivo studies suggested that the reduction of lipid accumulation appeared to be associated with the mitigation of oxidative stress [11,12,13]. Thus, there is a need to develop antioxidants, especially from natural sources, which may efficiently attenuate adipocyte lipid accumulation.

Fucoidan represents a group of l-fucose-enriched sulfated polysaccharides extracted from brown seaweeds such as *Sargassum* spp. and *Fucus* spp. that have a worldwide distribution both in the sea and in the intertidal zone. Algal fucoidan has been characterized by a wide variety of biological activities, including antioxidant, antivirus, anti-inflammatory, antitumor, and antithrombotic and anticoagulant effects [14,15]. The biological activities of fucoidan are closely related to their molecular structures, which include fucose linkage, sugar type, sulfate content, and molecular weight. Among these factors, molecular weight is one of the most important factors determining the biological activities of polysaccharides [16]. High molecular weight polysaccharides may cause low solubility and processability, thereby hampering their penetration into the cell to perform a given function. In contrast, low-molecular-weight (LMW) sulfated polysaccharides show higher biological functions such as anticancer, antioxidant, and anticoagulation activities [4,17].

This study builds upon the work of our previous research [18,19]. Briefly, an oven-dried brown seaweed *S. crassifolium* was subjected to compressional-puffing at 18.3 kg/cm^2^, depigmentation by 95% ethanol, and extraction of fucoidan by hot water. The high temperature and high pressure compressional-puffing process (CPP) could primarily decompose the cellular matrix of algae and increase the extraction yields of fucoidan from brown seaweed [18]. The recovered crude extract of fucoidan was utilized to investigate LMW fucoidans with respect to different degradation reagent treatments, and the recovered LMW fucoidans were examined to determine their composition, structure, molecular weight, and biological functions, including antibacterial and antioxidant capacities as well as attenuation of lipid accumulation in 3T3-L1 adipocytes. To the best of our knowledge, no such studies have been reported in the literature relating to the evaluations of the antibacterial and attenuation of lipid accumulation in 3T3-L1 adipocytes caused by LMW fucoidans obtained from compressional-puffing-pretreated *S. crassifolium*. In addition, we aimed to explore the potential of LMW fucoidans as natural antibacterial and anti-adipogenic agents for use in the food, cosmetic, and nutraceutical industries.

## 2. Results and Discussion

### 2.1. Preparation of LMW Fucoidans

**Figure 1:**
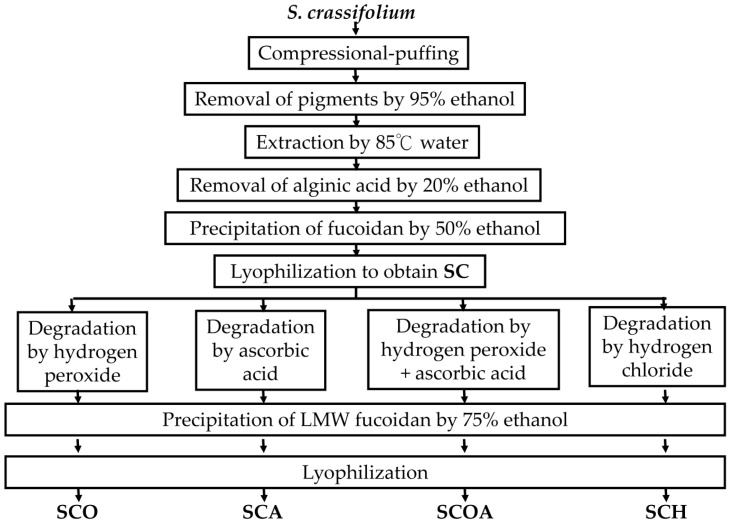
Flowchart of the preparation of SC (crude extract of fucoidan), SCO (degradation by hydrogen peroxide), SCA (degradation by ascorbic acid), SCOA (degradation by hydrogen peroxide + ascorbic acid), and SCH (degradation by hydrogen chloride) from *Sargassum* *crassifolium*.

The sample of *S. crassifolium* used in this study was composed of 2.36% protein, 0.98% lipid, 33.98% ash, and 62.67% carbohydrate (dry basis) [19]. Before extraction of fucoidan, the algal sample was pretreated by CPP with a steam pressure of 18.3 kg/cm^2^. The CPP has been proven to effectively increase the extraction yields of fucoidan from brown seaweeds [18] and to augment the extraction yields of total phenolics and total flavonoids from pine needles [20,21]. Afterwards, we obtained one fucoidan extract (namely, SC) from the compressional-puffed algal sample by 85 °C water extraction and ethanol precipitation (Figure 1). The extraction yields of fucoidan for compressional-puffed *S. crassifolium* and non-compressional-puffed *S. crassifolium* were 3.60% ± 0.11% and 0.68% ± 0.01% (*w*/*w*, dry basis), respectively, which suggests that CPP could increase more than five-fold the extraction yield of fucoidan. The fucoidan extract (SC) from compressional-puffed *S. crassifolium* was used for further degradation experiments. Various degradation reagents including hydrogen peroxide, ascorbic acid, hydrogen peroxide + ascorbic acid, and hydrogen chloride were utilized to degrade SC, and four LMW fucoidans, namely SCO, SCA, SCOA, and SCH, were obtained, respectively. A detailed presentation of the preparation processes for SC, SCO, SCA, SCOA, and SCH is provided in Figure 1.

### 2.2. Physicochemical and Compositional Analyses of Native and LMW Fucoidans

**Figure 2:**
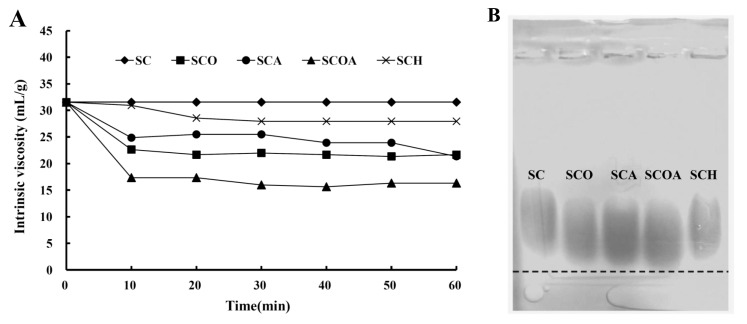
(**A**) The change of viscosity for SC, SCO, SCA, SCOA, and SCH at different time intervals; and (**B**) agarose gel electrophoresis analysis of SC, SCO, SCA, SCOA, and SCH.

**Figure 3:**
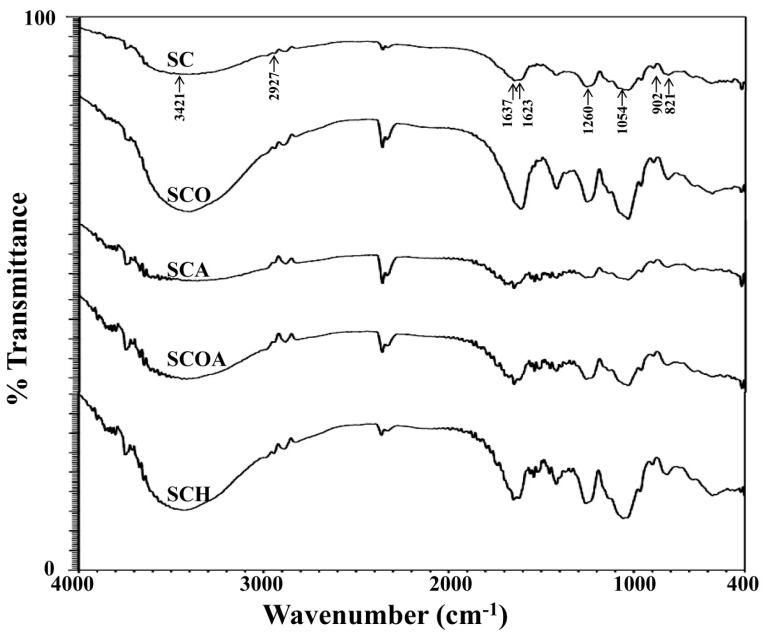
FTIR (Fourier Transform Infrared) spectra for SC, SCO, SCA, SCOA, and SCH.

To evaluate whether the degradation reagents successfully degraded fucoidan, the intrinsic viscosity of polysaccharide solution was analyzed and agarose gel electrophoresis of polysaccharide was performed. Previous studies indicated that the degradation of polysaccharide solution could result in a decrease of its intrinsic viscosity [22]. Figure 2A shows the change of viscosity of polysaccharide solution under the effects of different degradation reagents. It can be seen that the viscosities of degradation reagent-treated group (SCO, SCA, SCOA, and SCH) showed an obvious decrease as compared to the non-degraded sample (SC). The results suggest that all degradation reagents can successfully degrade SC. In addition, SCOA possessed the lowest intrinsic viscosity, indicating that the degradation reagent (hydrogen peroxide + ascorbic acid) might be the most efficient for degrading fucoidan and obtaining fucoidan with lower molecular mass. Figure 2B shows the electrophoretic mobility of the polysaccharide samples on agarose gel. Normally, a polysaccharide with lower molecular mass has higher mobility on agarose gel. Consistent with the result of the viscosity analysis (Figure 2A), SCOA (degraded by hydrogen peroxide + ascorbic acid) had the highest mobility on agarose gel, which indicates that SCOA may possess the lowest molecular mass. The molecular weights and molecular weight distributions for SC, SCO, SCA, SCOA, and SCH were further analyzed by high-performance liquid chromatography (HPLC) gel filtration analysis. Data presented in Table 1 revealed that the average molecular weight of extracts for SC was 600.23 kDa (peak area = 100%); for SCO were 608.41 kDa (peak area = 0.41%), 10.80 kDa (peak area = 39.61%), and 5.05 kDa (peak area = 59.98%); for SCA were 606.54 kDa (peak area = 0.90%), 14.89 kDa (peak area = 51.64%), and 6.87 kDa (peak area = 47.46%); for SCOA were 583.66 kDa (peak area = 0.47%), 13.35 kDa (peak area = 70.55%), and 7.62 kDa (peak area = 28.98%); and for SCH were 645.03 kDa (peak area = 20.11%) and 22.50 kDa (peak area = 79.89%). The data from gel filtration analysis provide evidence that, among these LMW fucoidans, SCO, SCA, and SCOA had a lower molecular weight as compared to that of SCH. For SCO, SCA, and SCOA, it was found that more than 99% of their molecular weights were less than 14.89 kDa. Moreover, SCO seemed to have the lowest molecular weight among these tested LMW fucoidans; however, the differences in the molecular weight distribution profiles among SCO, SCA, and SCOA were unremarkable. Previous studies showed that, besides molecular weight, the biological activities of fucoidan are also closely related to their compositions, such as the sugar type, monosaccharide composition, and sulfate content [15]. Thus, we measured the chemical composition and monosaccharide composition of SC, SCO, SCA, SCOA, and SCH, and the data are presented in Table 1. The total sugar contents for SCO, SCA, SCOA, and SCH ranged from 41.44% ± 2.36% to 53.17% ± 2.22% (*w*/*w*, dry basis), which were similar to that of SC (46.51% ± 1.28%). The results suggest that the degradation reagents would not obviously alter the total sugar content of fucoidan. Previous studies suggested that algal polysaccharide with a higher uronic acid content may be positively related to its antioxidant activity [23]. Here, we found that the contents of uronic acid for SCO, SCA, SCOA, and SCH ranged from 23.71% ± 0.31% to 29.04% ± 1.01%, which were significantly higher than that of SC (14.29% ± 0.62%) (Table 1), indicating that the degradation process may facilitate the increase of uronic acid content in these LMW fucoidans. l-fucose was reported as the major sugar constituent in fucoidan [24]. The fucose contents for SCO, SCA, SCOA, and SCH were 24.53% ± 1.89%, 31.64% ± 1.91%, 33.20% ± 0.54%, and 30.53% ± 1.89%, respectively, which were in general higher than that of SC (25.87% ± 1.09%), suggesting that the degradation reagents may increase the fucose content of LMW fucoidans (Table 1). The sulfate content of fucoidan plays a critical role in the biological functions as previously noted by other investigators [25,26]. Therefore, we measured the sulfate contents for SCO, SCA, SCOA, and SCH and the percentages were 17.47% ± 1.35%, 22.37% ± 0.98%, 22.23% ± 1.09%, and 19.77% ± 1.01%, respectively, which were significantly higher than that of SC (15.12% ± 0.67%), suggesting that degradation reagents may also increase the sulfate content of fucoidan (Table 1). Consequently, it was expected that these LMW fucoidans (SCO, SCA, SCOA, and SCH) may exhibit higher biological activities, and thus further investigations are warranted. In addition, previous investigations indicated that crude extracts of fucoidan may contain a lot of impurities such as proteins and polyphenols, which are hard to eliminate [27]. In the present study, we found that impurities of polysaccharides caused by the presence of protein and polyphenols could be detected in SCO (2.08 + 1.18 = 3.26, g/100 g, dry basis), SCA (2.02 + 0.79 = 2.81, g/100 g, dry basis), SCOA (2.14 + 0.62 = 2.76, g/100 g, dry basis), and SCH (2.30 + 0.86 = 3.16, g/100 g, dry basis), and these values were similar to that of SC (2.10 + 1.01 = 3.11, g/100 g, dry basis), indicating that degradation reagents did not obviously affect the impurity content of LMW fucoidans. Meanwhile, constituents of neutral monosaccharide of the fucoidans were analyzed by ion chromatography. For all the tested samples, fucose, galactose, mannose, and glucuronic acid were the dominant sugar units, and the molar ratios were similar between SC and the LMW fucoidans (SCO, SCA, SCOA, and SCH). Other monosaccharides such as glucose, rhamnose, and xylose were also seen in the samples; in general, their molar ratios in LMW fucoidans (SCO, SCA, SCOA, and SCH) were less than that in SC. In addition, the Fourier transform infrared (FTIR) results of these fucoidans shown in Figure 3 suggest that the typical signals for sulfated polysaccharides were obtained. The absorption bands at 3421, 2927, and 1637 cm^−1^ represent the O-H, C-H, and O-C-O vibrations [28]. The peaks at 821 cm^−1^ and 1623 cm^−1^ correspond to the bending vibrations of C-O-S of sulfate and the carbonyl C=O vibrations in uronic acid [29,30]. The peak at 1260 cm^−1^ indicates the asymmetric stretching vibration of the sulfate group (S=O) [22]. The peak at 902 cm^−1^ indicates β-pyranose ring vibration, and the peak at 1054 cm^−1^ shows C-O-H vibration [29,31]. Due to the similarity of the FTIR spectra in the native and LMW fucoidans, it was deduced that the position of sulfate groups and the structural aspects of sulfated polysaccharide were not significantly influenced by the degradation process. These findings were also consistent with previous investigations [28,32]. Taken together, four degradation reagents including hydrogen peroxide, ascorbic acid, hydrogen peroxide + ascorbic acid, and hydrogen chloride could effectively degrade fucoidan into LMW fucoidan as revealed by the intrinsic viscosity of polysaccharide solution, agarose gel electrophoresis of polysaccharide, and molecular weight analyses. The LMW fucoidans had higher fucose content, uronic acid content, and sulfate content than those of SC. However, the total sugar content, monosaccharide composition, and FTIR spectra between SC and LMW fucoidans were similar. Among SCO, SCA, SCOA, and SCH, the SCA and SCOA had relatively higher fucose content and higher sulfate content, and thus the biological functions of SCA and SCOA warrant further examination.

### 2.3. Antibacterial Activities of SC, SCO, SCA, SCOA, and SCH

**Figure 4:**
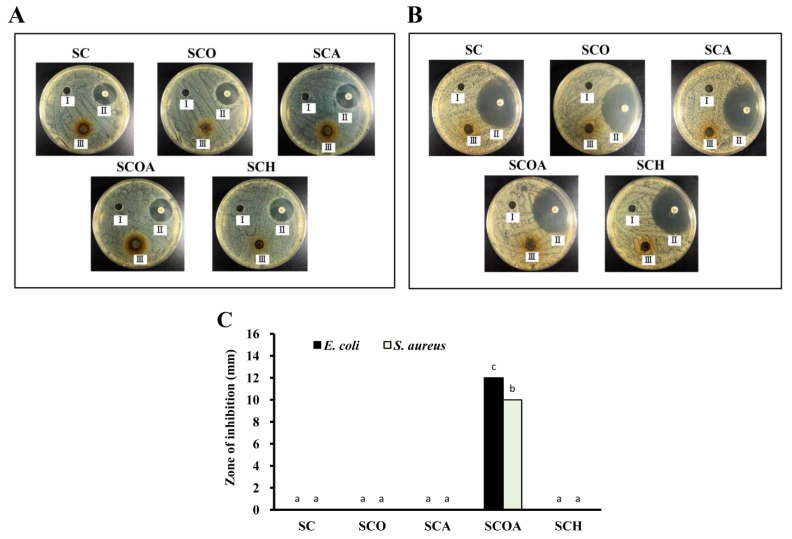
Zone of inhibition of: (**A**) *Escherichia coli*; and (**B**) *Staphylococcus aureus* for SC, SCO, SCA, SCOA, and SCH. I (acetate buffer), II (antibiotic), and III (polysaccharide sample) are shown in each dish; (**C**) The bar graph summarizes the three separate antibacterial experiments and shows the zone of inhibition according to treatments. Results are shown as mean ± SD of three independent experiments. Different letters (in a, b, and c) mean statistically significant differences at *p* < 0.05. Differences exist between columns labeled with different letters, *p* < 0.05.

Previous investigations indicated that fucoidans from *Laminaria japonica* showed no antibacterial activity before depolymerization; however, their depolymerized products could effectively inhibit the proliferation of *Escherichia coli* and *Staphylococcus aureus* [33]. Here, we evaluated the antibacterial properties of SC, SCO, SCA, SCOA, and SCH against one Gram-negative bacterium (*E. coli*) and one Gram-positive bacterium (*S. aureus*). As seen in Figure 4, SC showed no antibacterial activity against the tested bacteria. Moreover, for the LMW fucoidans, only SCOA exhibited antibacterial activity against *E. coli* and *S. aureus*. There are two possible antibacterial pathways of sulfated polysaccharides that have been proposed in previous studies [33,34,35]. One pathway involves the binding of polysaccharides with the bacterial surface resulting in destruction of the cell membrane, which leads to the leakage of proteins and essential nutrients, and eventually causes the death of cells. The other pathway is thought to involve the trapping of nutrients (such as cationic minerals in the culture medium) by the negatively charged sulfated polysaccharides, leading to reduced bioavailability of the nutrients. In the present study, SCOA showed apparent antibacterial activity against Gram-negative *E. coli* and Gram-positive *S. aureus* as compared to other LMW fucoidans, and we speculate that the reason may be attributed to its ability to trap cationic minerals. However, more evidence is needed to elucidate this mechanism. In summary, SCOA exhibits antibacterial properties and has the potential for application as an effective alternative to antibiotics in the fields of food processing, agriculture, biomedicine, and other industries.

### 2.4. Antioxidant Activities of of SC, SCO, SCA, SCOA, and SCH

**Figure 5:**
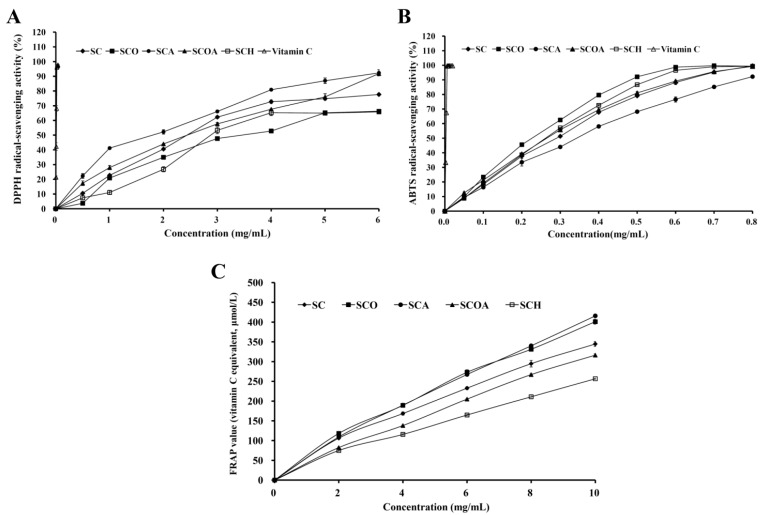
Antioxidant activities of SC, SCO, SCA, SCOA, and SCH: (**A**) DPPH radical-scavenging activity for SC, SCO, SCA, SCOA, SCH, and vitamin C; (**B**) ABTS radical-scavenging activity for SC, SCO, SCA, SCOA, SCH, and vitamin C; and (**C**) FRAP value for SC, SCO, SCA, SCOA, and SCH. Results are shown as mean ± SD of three independent experiments.

The antioxidant activities of SC, SCO, SCA, SCOA, and SCH were examined by 2,2-diphenyl-1-picrylhydrazyl (DPPH), 2,2′-azino-bis(3-ethylbenzothiazoline-6-sulfonic acid) diammonium salt (ABTS), and ferric reducing antioxidant power (FRAP) analyses. DPPH is widely used to evaluate antioxidant activity in a comparatively short time [4]. Figure 5A shows the DPPH radical-scavenging properties of SC, SCO, SCA, SCOA, SCH, and vitamin C (as a reference). It can be seen that all fucoidan samples displayed DPPH radical-scavenging activity in a dose-dependent pattern. Among LMW fucoidan samples, SCA and SCOA exhibited higher DPPH radical-scavenging activity. The IC_50_ values (concentration of fucoidan capable of scavenging 50% of DPPH) of the fucoidans (SC, SCO, SCA, SCOA, and SCH) on DPPH radical-scavenging activity were observed to be 2.44 ± 0.00, 3.45 ± 0.03, 1.81 ± 0.09, 2.43 ± 0.07, and 2.88 ± 0.06 mg/mL, respectively (Figure 5A), in which the potency of DPPH radical-scavenging activity among these fucoidans was: SCA > SCOA ≈ SC > SCH > SCO (*p* < 0.05). Wang et al. (2010) reported that the IC_50_ of the DPPH radical-scavenging activity for LMW fucoidan extracted from *L.* *japonica* was about 3.7 mg/mL [4], which was inferior to our result. ABTS radical decolorization assay works on a mechanism based on the decolorization of ABTS^•^^+^ when it reacted with hydrogen-donating antioxidant [36]. The ABTS^•^^+^ scavenging properties of SC, SCO, SCA, SCOA, SCH, and vitamin C (as a reference) are presented in Figure 5B. It was found that all fucoidans displayed ABTS^•^^+^ scavenging activity in a dose-dependent manner. Among LMW fucoidans, the SCO and SCH exhibited higher ABTS^•^^+^ scavenging activity. The FRAP assay measures the antioxidant effect of any substance in the reaction medium as reducing ability. The antioxidant potential of the samples was estimated from their ability to reduce TPTZ-Fe(III) complex to TPTZ-Fe(II) complex [37]. The FRAP antioxidant activity of SC, SCO, SCA, SCOA, and SCH are presented in Figure 5C. It can be seen that all fucoidan samples displayed FRAP antioxidant activity in a dose-dependent manner. Among LMW fucoidans, the SCA and SCO exhibited higher FRAP antioxidant activity. In addition, the FRAP value of SCA was observed to be 41.59 ± 0.40 μmol of vitamin C/g of fucoidan extract, which was superior to the FRAP value (18.36 ± 0.11 μmol of vitamin C/g of fucoidan extract) of fucoidan extracted from *S.* *hemiphyllum* performed by our laboratory. Taken together, these results confirmed that all LMW fucoidans exhibited antioxidant capacities. Lipid accumulation in adipocytes plays a critical role in the pathogenesis of obesity and related metabolic disorders [9,10], and it has also been shown to be associated with oxidative stress [11,12,13]. Since all LMW fucoidans exhibited antioxidant capacity, further studies are needed to fully elucidate their anti-adipogenic functions.

### 2.5. Attenuation of Lipid Accumulation in 3T3-L1 Adipocytes by SC, SCO, SCA, SCOA, and SCH

**Figure 6:**
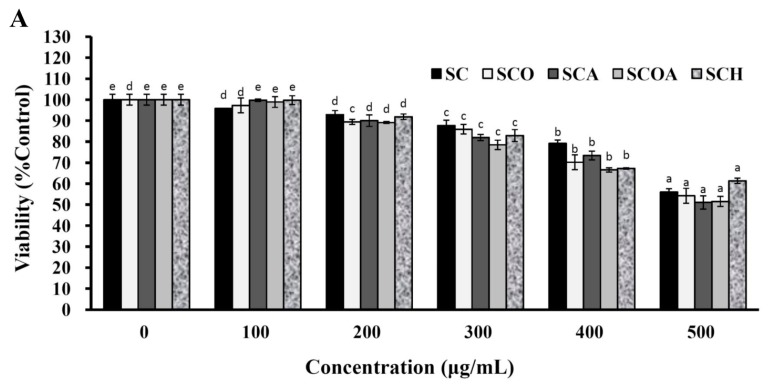

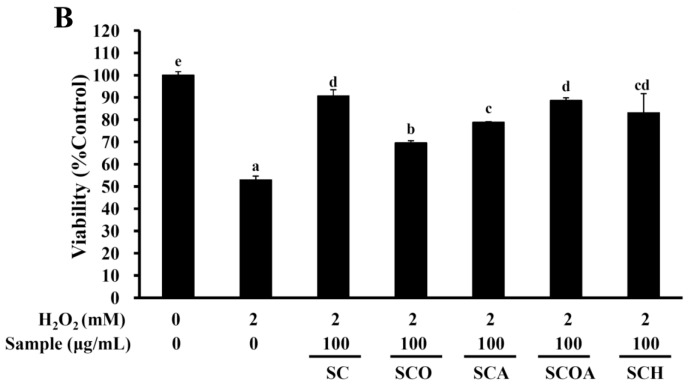
Effects of SC, SCO, SCA, SCOA, and SCH and H_2_O_2_ treatment with or without SC, SCO, SCA, SCOA, and SCH pretreatment on cell viability of 3T3-L1 cells: (**A**) 3T3-L1 preadipocytes were treated with SC, SCO, SCA, SCOA, or SCH (0–500 μg/mL) for 48 h, and cell viability was assessed. Results are shown as mean ± SD of three independent experiments. In each group of columns related to each concentration of sample, differences exist between columns labeled with different letters, *p* < 0.05; (**B**) 3T3-L1 adipocytes were pretreated with 100 μg/mL SC, SCO, SCA, SCOA, or SCH, followed by treatment with 2 mM H_2_O_2_ for 24 h, and cell viability was assessed. Results are shown as mean ± SD of three independent experiments. Different letters (in a, b, c, d, and e) mean statistically significant differences at *p* < 0.05. In each group of columns related to each treatment, differences exist between columns labeled with different letters, *p* < 0.05.

**Figure 7:**
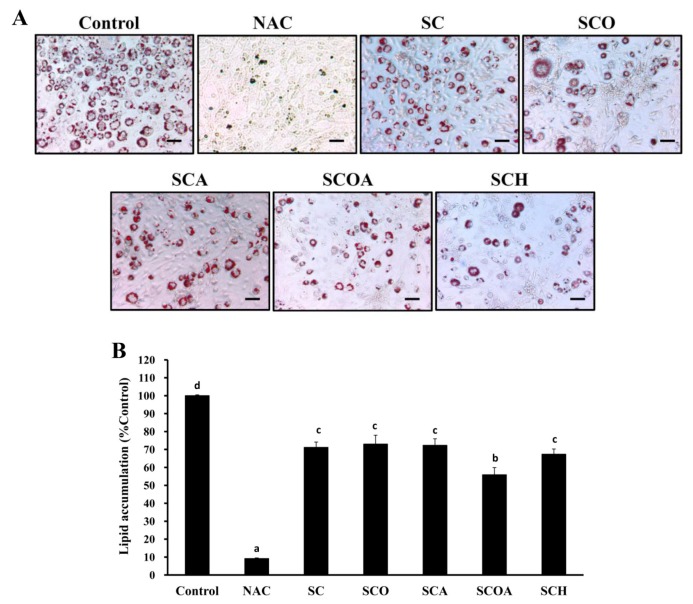
Effects of SC, SCO, SCA, SCOA, SCH, and NAC on lipid accumulation during the differentiation of 3T3-L1 preadipocytes: (**A**) 3T3-L1 preadipocytes were treated without or with SC, SCO, SCA, SCOA, SCH (100 μg/mL) and NAC (10 mM), and differentiating 3T3-L1 cells with visible lipid accumulation were examined on Day 8 by Oil Red O staining. The scale bar (100 μm) is shown in each graph; (**B**) The bar graph summarizes the three separate experiments and shows the percentage of lipid accumulation in 3T3-L1 adipocytes according to treatments. Results are shown as mean ± SD of three independent experiments. Different letters (in a, b, c, and d) mean statistically significant differences at *p* < 0.05. Differences exist between columns labeled with different letters, *p* < 0.05.

With respect to in vitro adipocyte differentiation, 3T3-L1 is a well-known and frequently used preadipocyte cell line for studies of function and adipose tissue accumulation [38]. 3T3-L1 cells undergo adipogenic differentiation when treated with a cocktail comprising dexamethasone (DEX), methylisobutylxanthine, and insulin. After acquisition of adipocyte function and morphology, 3T3-L1 cells can accumulate microscopically detectable triglyceride droplets [39]. To evaluate the cytotoxic effect of SC, SCO, SCA, SCOA, and SCH on 3T3-L1 preadipocytes, the cells were treated with different concentrations of SC, SCO, SCA, SCOA, and SCH for 48 h, and then the cell viability of 3T3-L1 preadipocytes was evaluated by the MTT (3-(4,5-dimethylthiazol-2-yl)-2,5-diphenyltetrazolium bromide) assay. As shown in Figure 6A, at a concentration of 100 μg/mL, none of the studied LMW fucoidans exhibited apparent cytotoxicity to 3T3-L1 preadipocytes. Therefore, the concentration of 100 μg/mL for LMW fucoidans was utilized for further cellular experiments. H_2_O_2_ is known to be a major source of ROS and an apoptosis inducer in various cell types [19,40]. Thus, H_2_O_2_ was used to evaluate the protective effects of LMW fucoidans against oxidation in 3T3-L1 adipocytes. The treatment of 3T3-L1 adipocytes with 2 mM H_2_O_2_ for 24 h decreased the cell viability to 52.95% ± 1.75% compared with the control group (Figure 6B). Moreover, pretreatment of 3T3-L1 adipocytes with SC, SCO, SCA, SCOA, or SCH at a concentration of 100 μg/mL followed by treatment with 2 mM H_2_O_2_ for 24 h attenuated H_2_O_2_-induced cellular cytotoxicity, and the cell viability values were 90.66% ± 2.83%, 69.55% ± 0.96%, 78.80% ± 0.27%, 88.63% ± 1.24%, and 83.06% ± 8.70% compared with the control group, respectively (Figure 6B). Owing to their antioxidant properties (Figure 5), LMW fucoidans could effectively reverse the H_2_O_2_-induced cellular cytotoxicity in 3T3-L1 adipocytes. Among the tested LMW fucoidans, SC and SCOA had the highest activity for reversal of H_2_O_2_-induced cell death in 3T3-L1 adipocytes. Lipid accumulation in adipocytes may cause adipocyte hypertrophy and accumulation of adipose tissue, leading to obesity and metabolic syndrome [9,10]. To quantify the lipid accumulation in 3T3-L1 adipocytes, an Oil Red O staining method can be used. As shown in Figure 7A,B, when 3T3-L1 cells were treated with N-acetyl cystein (NAC) (as a positive control), SC, SCO, SCA, SCOA, or SCH during the differentiation of preadipocytes to adipocytes, the lipid accumulation was decreased by 90.85%, 28.82%, 27.00%, 27.65%, 44.14%, and 32.66%, respectively. These results clearly suggest that SC, SCO, SCA, SCOA, and SCH exhibited anti-adipogenic effects. Among the LMW fucoidans tested, SCOA showed the highest effect on attenuation of lipid accumulation in 3T3-L1 adipocytes. We speculate that this phenomenon may be explained by the higher fucose and sulfate contents in SCOA. However, further in vitro experiments such as oversulfation of LMW fucoidan and in vivo studies are warranted. Few studies on the inhibition of lipid accumulation in 3T3-L1 adipocytes by LMW fucoidans have been reported. Therefore, we compared the inhibitory effect of our LMW fucoidans on differentiation of 3T3-L1 preadipocytes with that of fucoidans from other investigations. The inhibitory effect of fucoidan on differentiation of 3T3-L1 preadipocytes varied considerably among different reports. Park et al. (2011) utilized a commercially available high purity fucoidan (extracted from *Focus vesiculosus* and obtained from Sigma) and found it could decrease the lipid accumulation of 3T3-L1 adipocytes by 16.5% at a concentration of 100 μg/mL [41], which was inferior to our result for SCOA at the same concentration. However, Kim et al. (2010) utilized a commercially available high purity fucoidan (provided by Sigma) and found it could decrease the lipid accumulation of 3T3-L1 adipocytes by 50.5% at a concentration of 100 μg/mL [42], which was similar to the result of our SCOA at the same concentration. These findings suggest that our LMW fucoidans, especially SCOA had a similar inhibitory effect on lipid accumulation to that of highly purified fucoidan. Since highly purified fucoidan requires complicated extraction and purification processes and high production cost, our SCOA may be a good alternative for inhibition of lipid accumulation. In summary, none of the LMW fucoidans showed any obvious cytotoxicity to 3T3-L1 preadipocytes at a concentration of 100 μg/mL. The LMW fucoidans could effectively reverse the H_2_O_2_-induced cellular cytotoxicity of 3T3-L1 adipocytes. In addition, all LMW fucoidans exhibited an anti-adipogenic effect as revealed by the attenuation of lipid accumulation during differentiation of 3T3-L1 preadipocytes. Among the LMW fucoidans tested, SCOA showed the highest effect on attenuation of lipid accumulation, and thus it may have potential as a natural and safe anti-adipogenic agent for food and nutraceutical applications.

## 3. Materials and Methods

### 3.1. Materials and Chemicals

A sample of *S. crassifolium* (SC) was collected from Pingtung, Taiwan and then washed with fresh water, oven-dried at 50 °C, and kept in plastic bags at 4 °C until further experiments. l-fucose, l-rhamnose, d-glucose, d-glucuronic acid, d-galactose, d-mannose, d-xylose, d-galacturonic acid, gallic acid, sodium carbonate, DPPH, ABTS, 2,4,6-tripyridyl-s-triazine (TPTZ), 3-isobutyl-1-methylxanthine (IBMX), ammonium acetate, Bradford reagent, bovine serum albumin (BSA), DEX, insulin, NAC, nitroblue tetrazolium (NBT), Oil Red O, thioglycolic acid solution, toluidine blue, and dimethyl sulfoxide (DMSO) were purchased from Sigma-Aldrich (St. Louis, MO, USA). Potassium bromide (KBr) was purchased from Merck (Darmstadt, Germany). 2,2,2-Trifluoroacetic acid (TFA) was obtained from Panreac (Barcelona, Spain). Peptone, TSA, and TSB were purchased from BD Biosciences (San Jose, CA, USA). DMEM, trypsin/EDTA, fetal calf serum (FCS), penicillin, and streptomycin were purchased from Gibco Laboratories (Grand Island, NY, USA). All other reagents were of analytical grade or the best grade available.

### 3.2. Compressional-Puffing Procedure

The dried algal sample was puffed according to our previously described procedure [18] with slight modification. In brief, the algal sample was automatically put into the chamber and the puffing conditions were set with temperature at 220 °C. After CPP, the algal sample was ground into fine particles and stored at 4 °C for further extraction experiments.

### 3.3. Water Extraction Procedure

We followed the methods of Yang et al. (2017) [19]. In brief, the compressional-puffed algal sample was mixed with 95% ethanol (*w*/*v* = 1:10), shaken for 4 h at room temperature to remove pigments, proteins and lipid, and then centrifuged at 970× *g* for 10 min. The residue was collected, mixed with double-distilled water (*w*/*v* = 1:10) and placed in a water-bath kept at 85 °C for 1 h with shaking (120 rpm) to extract the polysaccharides. The mixture was centrifuged at 3870× *g* for 10 min and the supernatant was collected. Ethanol (95%) was added into the supernatant to give a final ethanol concentration of 20% in order to precipitate alginic acid. The mixture was centrifugated at 9170× *g* for 30 min, the supernatant was collected, and 95% ethanol was added until a final ethanol concentration of 50% was reached in order to obtain fucoidan precipitate. The ethanol-precipitated fucoidan was then recovered by centrifugation at 9170× *g* for 30 min and lyophilized. Extraction yield was calculated using the following equation:

Extraction yield (%) = (*g*_A_/*g*_B_) × 100 (1)

where *g*_A_ represents the weight of the extracted solid on a dry basis, and *g*_B_ is the weight of the sample on a dry basis.

### 3.4. Degradation Procedure

A solution of native fucoidan sample (0.1 g) in double distilled water (10 mL) was added in 10 mM H_2_O_2_, 10 mM ascorbic acid, a mixed solution of 10 mM H_2_O_2_ and 10 mM ascorbic acid, or 0.1 N HCl, respectively. For HCl treatment, the pH of reaction solution was adjusted to 2.0 by 0.1 N NaOH at the start of reaction. Then the solution was stirred to start the degradation reaction. The time of reaction was 1 h. For HCl treatment, the pH of reaction solution was adjusted to 7.0 by 0.1 N NaOH at the end of reaction. After the reaction, the LMW fucoidan was precipitated by adding 75% ethanol and the ethanol-precipitated LMW fucoidan was then recovered by centrifugation at 9170× *g* for 30 min and lyophilized.

### 3.5. Chemical Methods

The phenol-sulfuric acid colorimetric method was used to determine the total sugar content, and d-galactose was used as the standard [43]. The fucose content was determined by the method of Gibbons [44], and l-fucose was used as the standard. Uronic acids were estimated by the colorimetric method using d-galacturonic acid as the standard [45]. Protein was quantified by the Bradford method using BSA as the standard [46]. Polyphenols were analyzed by the Folin–Ciocalteu method and gallic acid was used as the standard [47]. Sulfate content of polysaccharide was determined by first hydrolyzing the polysaccharide with 1 N HCl solution for 5 h at 105 °C. The hydrolysate was then quantified based on the percentage of sulfate composition using Dionex ICS-1500 Ion Chromatography (Sunnyvale, CA, USA) with an IonPac AS9-HC column (4 × 250 mm) at a flow rate of 1 mL/min at 30 °C with conductometric detection. The eluent was 9 mM Na_2_CO_3_, and K_2_SO_4_ was utilized as the standard.

### 3.6. Analysis of Monosaccharide Composition

The monosaccharide composition of polysaccharide was analyzed according a previously described method [18], using l-fucose, d-xylose, d-galactose, d-glucose, d-glucuronic acid, l-rhamnose, and d-mannose as the standards.

### 3.7. Intrinsic Viscosity Analysis

The intrinsic viscosity of polysaccharide solution was determined in an Ubbelohde viscosimeter at 25 °C. The result of intrinsic viscosity (η_r_) was calculated using the following equation:

η_r_ = (ln t/t_0_)/c (2)

where t is the solution flow time (s), t_0_ is the solvent flow time (s), and c is the concentration of solution (g/mL) [22].

### 3.8. Agarose Gel Electrophoresis

About 5 μg of polysaccharide sample was applied to a 0.5% agarose gel in 0.05 M 1,3-diaminopropane/acetate buffer (pH 9.0) for 1 h at 110 V. The polysaccharide in the gel was fixed with 0.1% *N*-cetyl-*N*,*N*,*N*-trimethylammonium bromide in water. Thereafter, polysaccharides were stained using 0.1% toluidine blue in acetic acid/ethanol/water (0.1:5:5, *v*/*v*/*v*) [48].

### 3.9. Fourier Transform Infrared (FTIR) Spectroscopy

The FTIR analysis was performed according to the method of Huang [49]. In brief, polysaccharide and KBr (*w*/*w*, 1:50) were mixed and ground evenly until particles measured less than 2.5 μm in size. The transparent KBr pellets were made at 500 kg/cm^2^ under vacuum. The FTIR spectra were obtained using a FT-730 spectrometer (Horiba, Kyoto, Japan), and the absorbance was read between 400 and 4000 cm^−1^. Pellet of KBr alone was used as a background.

### 3.10. Molecular Weight Analysis

The molecular weight analysis of the polysaccharides was conducted according to the method of Yang [19]. The standards used to calibrate the column were various dextrans with different molecular weights (1, 5, 12, 50, 150, and 670 kDa), which were obtained from Sigma-Aldrich (Sigma-Aldrich, St. Louis, MO, USA)

### 3.11. Zone of Inhibition

Two bacteria were tested for antibacterial activity of polysaccharides. These included one Gram-negative bacterium (*E. coli* BCRC 10675) and one Gram-positive bacterium (*S. aureus* BCRC 10780), which were obtained from the Culture Collection and Research Center of the Food Industry Research and Development Institute, Hsinchu, Taiwan. Antibacterial activity was measured using a previously described method [50]. In brief, *E. coli* and *S. aureus* were grown in tryptic soy broth (TSB) medium (Difco Laboratories, Detroit, MI, USA) for 24 h at 30 °C, and 0.1 mL of each culture of bacteria at the proper cell density was spread on Tryptic soy agar (TSA, Difco Laboratories, Detroit, MI, USA) plate surfaces. A volume measuring 100 μL of each polysaccharide sample (400 mg/mL in 0.05 M acetate buffer, pH 6.0) or 0.05 M acetate buffer (as a negative control) was delivered separately into wells (8 mm in diameter). Positive control paper disc was prepared by impregnating it with a volume measuring 50 μL of 10% (*v*/*v*) Antibiotic-Antimycotic Solution (containing 10,000 units/mL penicillin, 10 mg/mL streptomycin, and 25 µg/mL amphotericin) (Corning, Corning, NY, USA). The plates were incubated at 35 °C for 24 h. After 24 h, the antibacterial activity of polysaccharides against the test bacteria was observed to determine whether any zones of microbial growth inhibition had developed, which would indicate the occurrence of antimicrobial action, and any such activity was expressed in terms of average diameter of the zone of inhibition measured in millimeters.

### 3.12. DPPH Radical Scavenging Activity

The DPPH radical scavenging activity was determined according to a method described elsewhere [19]. Briefly, 50 µL of sample was added to 150 µL 0.1mM freshly prepared DPPH solution (in methanol). The mixture was shaken vigorously for 1 min, left to stand for 30 min in the dark at room temperature, and the absorbance of all sample solutions was measured at 517 nm using an ELISA reader (PowerWave 340, Bio-Tek Instruments, Winooski, VT, USA). The radical-scavenging activity was calculated using the following equation:

(3) $${\mathrm{DPPH}}_{\text{radical-scavenging}}\;(\%)=\left[1-\frac{A_{\mathrm{sample}}}{A_{\mathrm{control}}}\right]\times 100$$

where A_sample_ is the absorbance of the methanol solution of DPPH with tested samples, and A_control_ represents the absorbance of the methanol solution of DPPH without the sample.

### 3.13. ABTS Radical Cation Scavenging Activity

The scavenging activity of the samples against ABTS radical cation was measured according to a method described elsewhere [19]. In brief, the ABTS^•^^+^ solution was prepared by reacting 5 mL of ABTS solution (7 mM) with of 88 µL of potassium persulfate (140 mM), and the mixture was kept in the dark at room temperature for 16 h. The solution was diluted with 95% ethanol to obtain an absorbance of 0.70 ± 0.05 at 734 nm. To start the assay, 100 µL diluted ABTS^•^^+^ solution was mixed with 100 µL of various sample solutions. The mixture was allowed to react at room temperature for 6 min, and the absorbance of all sample solutions at 734 nm was measured using an ELISA reader (PowerWave 340, Bio-Tek Instruments, Winooski, VT, USA). The blank was prepared in the same manner, except that distilled water was used instead of the sample. The activity of scavenging ABTS^•^^+^ was calculated according to the following equation:

(4) $${\mathrm{ABTS}}_{\mathrm{cation}\;\text{radical-scavenging}}\;(\%)\;=\left[1-\frac{A_{\mathrm{sample}}}{A_{\mathrm{control}}}\right]\times 100$$

where A_sample_ is the absorbance of ABTS with tested samples, and A_control_ represents the absorbance of ABTS without the sample.

### 3.14. FRAP Assay

The FRAP assay was performed according to the protocol proposed by Benzie and Strain [51] Briefly, the FRAP solution was prepared by adjusting 10 mL of acetate buffer (300 mM) to pH 3.6 via the addition of acetic acid. It was then mixed with 1 mL of ferric chloride hexahydrate (20 mM) dissolved in distilled water and 1 mL of TPTZ (10 mM) dissolved in HCl (40 mM). A volume measuring 50 μL of the test sample dissolved at different concentrations was prepared. Freshly prepared FRAP solution (450 μL) warmed at 37 °C was added to the sample and allowed to react for 30 min under dark conditions, while the same volume of acetate buffer was utilized as the blank. The absorbance at 593 nm was monitored by an ELISA reader (PowerWave 340, Bio-Tek Instruments, Winooski, VT, USA). Vitamin C was used as a standard and FRAP values were expressed as micromole vitamin C equivalents per gram of sample on a dry basis (μmol vitamin C/g sample, dry basis).

### 3.15. Adipocyte Cell Culture

Mouse 3T3-L1 (ATCC^®^ CCL-92.1™) cells were purchased from the Food Industry Research and Development Institute, Hsinchu, Taiwan which had originally obtained them from the American Type Culture Collection (ATCC) (Rockville, CT, USA). Cultured 3T3-L1 cells were maintained in DMEM containing 10% FCS. To induce differentiation, two-day post-confluent preadipocytes were cultured in DMEM containing 10% FCS, 10 μg/mL insulin, 1 μM DEX, and 0.5 mM IBMX for three days. Cells were then cultured in DMEM supplemented with 10% FCS and 10 μg/mL insulin for another two days, after which cells were fed every other day with DMEM containing 10% FCS at 37 °C and 5% CO_2_, and thus the total duration of the differentiation process was 8 days [52].

### 3.16. Cell Viability Test

Cell viability was measured by quantitative colorimetric MTT assay [53]. Briefly, 3T3-L1 cells (1 × 10^6^/mL in a 96-well plate) were plated with culture medium and incubated for 48 h at 37 °C, with 5% CO_2_ in a humidified atmosphere. The cells were then incubated with test compounds at various concentrations for various times. The reaction was stopped by removing the treatment media, adding MTT reagent (1 mg/mL), and then allowing the reagent to react at 37 °C in 5% CO_2_ for 4 h. MTT was removed, and cells were lysed with DMSO. The absorbance at 570 nm was measured using an ELISA reader (PowerWave 340, Bio-Tek Instruments, Winooski, VT, USA). The cell viability (%) was calculated using the following equation:

(5) $$\mathrm{Cell}\;\mathrm{viability}\;(\%)=\left(\frac{T}{C}\right)\times 100$$

where T is the absorbance in the test, and C is the absorbance for the control.

### 3.17. Lipid Accumulation

Intercellular lipid accumulation was measured by Oil Red O staining during adipocyte differentiation [54]. In brief, treated mature adipocytes were washed with PBS and fixed for 1 h with 10% formalin (*v*/*v* in PBS). The Oil Red O solution was prepared by dissolving 0.5 g of Oil Red O powder in 100 mL of 100% isopropanol and diluted to a final volume of 167 mL with H_2_O. Each well was stained with dye solution for 15 min, after which the cells were washed with H_2_O four times and allowed to air-dry. Stained cells were visualized by light microscopy and photographed. The Oil Red O dye was eluted from the lipid droplets by adding 100% isopropanol for 5 min. The resulting eluent was analyzed by an ELISA reader (PowerWave 340, Bio-Tek Instruments, Winooski, VT, USA) at a wavelength of 490 nm. The lipid accumulation (%) was calculated using the following equation:

(6) $$\mathrm{Lipid}\;\mathrm{accumulation}\;(\%)=\left(\frac{A_{\mathrm{treatment}}-A_{\mathrm{blank}}}{A_{\mathrm{control}}-A_{\mathrm{blank}}}\right)\times 100$$

where A_treatment_ is the absorbance for the cells and test samples, A_control_ is the absorbance for the cells only, and A_blank_ is the absorbance for the medium only.

### 3.18. Statistical Analysis

Results are presented as means ± standard deviation (SD) of three independent experiments. Data were analyzed using the Statistical Package for the Social Sciences (SPSS). The results obtained were analyzed using one-way analysis of variance (ANOVA), followed by Duncan’s Multiple Range tests. Data were considered statistically different at *p* < 0.05.

## 4. Conclusions

In this paper, we extracted fucoidan from compressional-puffed *S. crassifolium* by hot water. Various degradation reagents were used to degrade the crude extract of fucoidan (SC) and four LMW fucoidans, SCO, SCA, SCOA, and SCH, were obtained. The LMW fucoidans had higher uronic acid content and sulfate content than those of SC. All LMW fucoidans showed antioxidant activities, as revealed by the DPPH, ABTS, and FRAP methods. It was found that only SCOA exhibited antibacterial activity. Additional biological experiments showed that SC and SCOA had relatively high activity for the reversal of H_2_O_2_-induced cell death in 3T3-L1 adipocytes, and SCOA showed the highest effect on attenuation of lipid accumulation in 3T3-L1 adipocytes. Among these LMW fucoidans, SCOA showed antibacterial activity and had a high fucose content, high sulfate content, high activity for the reversal of H_2_O_2_-induced cell death, and a marked effect on attenuation of lipid accumulation, and we thus recommend it as a natural and safe antibacterial and anti-adipogenic agent for food, cosmetic, and nutraceutical applications. Further in vivo anti-adipogenic studies, especially on SCOA, are required.

## Figures and Tables

**Table 1:** Composition and molecular weight analyses for SC, SCO, SCA, SCOA, and SCH.

Molecular Weight (MW)	SC	SCO	SCA	SCOA	SCH
Peak 1 (MW (kDa)/Peak area (%))	600.23/100.00	608.41/0.41	606.54/0.90	583.66/0.47	645.03/20.11
Peak 2 (MW (kDa)/Peak area (%))	ND ^3^	10.80/39.61	14.89/51.64	13.35/70.55	22.50/79.89
Peak 3 (MW (kDa)/Peak area (%))	ND	5.05/59.98	6.87/47.46	7.62/28.98	ND
Chemical composition	SC ^2^	SCO ^2^	SCA ^2^	SCOA ^2^	SCH ^2^
Total sugar (%) ^1^	46.51 ± 1.28 ^b^	45.84 ± 0.81 ^b^	41.44 ± 2.36 ^a^	53.17 ± 2.22 ^c^	50.57 ± 1.51 ^c^
Uronic acid (%) ^1^	14.29 ± 0.62 ^a^	27.71 ± 1.01 ^c^	27.38 ± 0.94 ^c^	29.04 ± 1.01 ^c^	23.71 ± 0.31 ^b^
Fucose (%) ^1^	25.87 ± 1.09 ^a^	24.53 ± 1.89 ^a^	31.64 ± 1.91 ^b^	33.20 ± 0.54 ^b^	30.53 ± 1.89 ^b^
Sulfate (%) ^1^	15.12 ± 0.67 ^a^	17.47 ± 1.35 ^b^	22.37 ± 0.98 ^c^	22.23 ± 1.09 ^c^	19.77 ± 1.01 ^b^
Protein (%) ^1^	2.10 ± 0.04 ^a^	2.08 ± 0.20 ^a^	2.02 ± 0.01 ^a^	2.14 ± 0.11 ^a b^	2.30 ± 0.04 ^b^
Polyphenols (%) ^1^	1.01 ± 0.13 ^c^	1.18 ± 0.06 ^d^	0.79 ± 0.03 ^b^	0.62 ± 0.08 ^a^	0.86 ± 0.06 ^b^
Monosaccharide composition (molar ratio)	SC	SCO	SCA	SCOA	SCH
Fucose	1	1	1	1	1
Galactose	0.39	0.45	0.36	0.36	0.35
Mannose	0.30	0.36	0.38	0.34	0.34
Glucuronic acid	0.22	0.22	0.20	0.20	0.19
Glucose	0.15	0.10	0.10	0.09	0.09
Rhamnose	0.20	0.10	0.04	0.05	0.05
Xylose	0.06	0.01	0.01	ND	0.02

^1^ Total sugars (%), uronic acid (%), fucose (%), sulfate (%), protein (%), and polyphenols (%) = (g/g_sample__, dry basis_) × 100; ^2^ Values are mean ± SD (*n* = 3); values in the same row with different letters (^a^, ^b^, ^c^, and ^d^) are significantly different (*p* < 0.05); ^3^ ND: not detected.
